# Beyond illness severity: transdiagnostic cognitive vulnerabilities as independent predictors of psychological distress in parents of children with rare genetic disorders

**DOI:** 10.3389/fpsyt.2026.1963031

**Published:** 2026-09-29

**Authors:** Goran Cuturilo, Tatjana Vukosavljevic Gvozden, Matija Gvozden, Nadja Cukanović, Borjanka Batinic

**Affiliations:** 1 Faculty of Medicine, University of Belgrade, Belgrade, Serbia; 2 University Children’s Clinic, Belgrade, Serbia; 3 Department of Psychology, Faculty of Philosophy, University of Belgrade, Belgrade, Serbia

**Keywords:** parental psychological distress, rare genetic disorders in children, parental intolerance of uncertainty, distress tolerance, tendency to worry

## Abstract

**Introduction:**

Parents of children with rare genetic disorders are at increased risk of depression, anxiety, and stress symptoms. While previous research has primarily focused on illness-related factors, less is known about the contribution of transdiagnostic cognitive vulnerabilities to parental psychological distress.

**Aim:**

This study examined whether intolerance of uncertainty, distress tolerance, and tendency to worry predict parental depression, anxiety, and stress symptoms beyond the severity of the child’s health status.

**Methods:**

The study included 184 parents (92 mother–father dyads) of children with rare genetic disorders and 94 parents of healthy children (14 mother–father dyads and 66 unpaired parents). Measures assessed child health status severity, intolerance of uncertainty, distress tolerance, tendency to worry, and symptoms of depression, anxiety, and stress.

**Results:**

Parents of children with rare genetic disorders, particularly mothers, reported higher levels of depression, anxiety, and stress symptoms compared with parents of healthy children. Greater severity of the child’s health condition was associated with higher levels of psychological distress. Lower distress tolerance and greater tendency to worry independently predicted depression, anxiety, and stress symptoms beyond the severity of the child’s health status.

**Conclusion:**

Transdiagnostic cognitive vulnerabilities, particularly lower distress tolerance and greater tendency to worry, together with female gender, were independently risk factors for parental psychological distress beyond the severity of the child’s health status severity. These findings suggest that preventive interventions and counseling programs for parents of children with rare genetic disorders should address not only illness-related burden but also modifiable cognitive vulnerabilities.

## 1 Introduction

Parents of children with rare genetic disorders represent a particularly vulnerable caregiver population, with an increased risk of developing emotional disorders such as depression, anxiety, and chronic stress (1, 2). Rare disorders (RD) are defined as conditions affecting fewer than 5 in 10,000 individuals (3). Although each disorder is rare on its own, taken together they affect approximately 6–10% of the population and include an estimated 6,000–8,000 conditions (4, 5). Most of these conditions emerge in childhood, and around 80% are genetic in origin (6, 7). This genetically determined subgroup of rare disorders, referred to as rare genetic disorders, typically comprises chronic and lifelong conditions involving developmental, neurological, and physical impairments that can significantly affect children’s quality of life and life expectancy (8). These disorders encompass a broad and clinically heterogeneous spectrum, including individuals with neurodevelopmental and/or genetic epilepsy disorders (e.g., Fragile X syndrome, Rett syndrome, or Dravet syndrome), progressive neurodegenerative or neuromuscular conditions (e.g., neuronal ceroid lipofuscinosis or Duchenne muscular dystrophy), chromosomal or monogenic syndromes associated with multiple congenital malformations (e.g., Down syndrome, 22q11.2 deletion syndrome, or Noonan syndrome), and other multisystem or sensory genetic disorders (e.g., neurofibromatosis type 1, tuberous sclerosis complex, or inherited disorders affecting hearing and vision).

Parenting a child with a rare genetic disorder brings a distinct set of challenges that often go far beyond typical caregiving demands. Parents are frequently faced with diagnostic uncertainty, unpredictable illness trajectories, limited treatment options, and relatively poor access to information and support (9). Together, these factors can create a substantial and ongoing psychological burden. At the same time, a consistent finding across studies is that higher levels of child disability are associated with greater parental distress, likely reflecting increased caregiving demands, reduced child independence, and persistent concerns about the child’s functioning and future (1, 2, 10). In line with this, parents of children with rare genetic disorders tend to report elevated caregiver burden, including higher levels of psychological distress and lower quality of life (11, 12). They are also at increased risk of emotional disorders (13–16), with a substantial proportion- between 39% and 67%- reporting clinically significant anxiety (13, 16). In most cases, caregiving responsibilities fall primarily on mothers, who therefore carry a disproportionate share of the burden and tend to report higher levels of anxiety and stress compared to fathers (17–21). Recent evidence suggests that this maternal disadvantage extends beyond psychological symptoms to broader caregiving, physical, occupational, and financial burdens. In rare neurogenetic conditions, mothers report greater caregiving burden and a stronger impact on physical health than fathers, while their well-being is additionally shaped by employment constraints, financial strain, and delays in obtaining a diagnosis (22, 23).

While illness-related factors, such as the severity of the child’s health condition, are clearly important and consistently associated with parental distress, they do not fully explain why some parents struggle more than others under similar circumstances (2). This suggests that additional psychological mechanisms may be at play. In recent years, transdiagnostic models of psychopathology have emphasized that emotional distress is shaped not only by exposure to stress, but also by relatively stable cognitive vulnerability factors that influence how individuals interpret and respond to stress (24–26). From this perspective, maladaptive responses to uncertainty, emotional discomfort, and repetitive negative thinking are seen as central processes in the development and maintenance of emotional disorders.

Among the transdiagnostic cognitive vulnerabilities, *intolerance of uncertainty* has emerged as one of the most extensively studied constructs, broadly defined as a tendency to interpret and respond negatively to uncertain situations (27–29). It has been linked to both anxiety and depression across different populations (30–33). In the context of rare genetic disorders, uncertainty is not merely occasional but pervasive, as parents often face unclear diagnoses, unpredictable disease trajectories, and uncertain long-term outcomes (34). For individuals high in intolerance of uncertainty, this may substantially amplify distress.

Closely related to intolerance of uncertainty is the *tendency to worry*, typically understood as a repetitive, negatively valanced cognitive process focused on potential future threats (35–37). Although worry can sometimes feel like a way of preparing for potential problems, it often turns into uncontrollable and unproductive “what if” thinking (38), which in turn increases vulnerability to anxiety and depression (39, 40). For parents of children with rare genetic disorders, ongoing concerns about complications, treatment outcomes, and future caregiving demands make tendency to worry particularly relevant.

Another potentially important vulnerability factor in this context is *distress tolerance*, defined as the ability to withstand negative emotional states (41). Higher distress tolerance is generally associated with better emotional outcomes and life satisfaction (42–45), whereas lower distress tolerance has been linked to emotional dysregulation and a range of psychological disorders, including anxiety and depression (46–49). In the context of chronic caregiving, ongoing medical demands, and disruptions in daily life, parents may be repeatedly exposed to situations that challenge their capacity to tolerate distress.

Although intolerance of uncertainty, worry, and distress tolerance are related transdiagnostic factors, they were selected for the present study because they capture theoretically distinguishable aspects of responding to the persistent uncertainty and emotional demands that characterize parenting a child with a rare genetic disorder. Within cognitive-behavioral models, intolerance of uncertainty is conceptualized as a relatively distal cognitive vulnerability, reflecting a negative disposition toward uncertainty and its perceived consequences, while on the other hand, worry represents a more proximal, repetitive, future-oriented cognitive process through which individuals attempt to anticipate or mentally manage potential threats (27, 29, 50). A recent meta-analysis of 115 studies found a mean association of r = .59 between intolerance of uncertainty and worry, while meta-analytic structural equation modeling supported a hierarchical model in which intolerance of uncertainty operates as a distal vulnerability and worry as a proximal cognitive process partially linking IU to anxiety (51). This distinction is also consistent with treatment research, showing that changes in intolerance of uncertainty can precede and account for subsequent reductions in worry (52). In this context, distress tolerance represents an additional and conceptually distinct dimension, encompassing the perceived capacity to withstand and function in the presence of aversive emotional states, rather than beliefs about uncertainty or repetitive thinking about possible future outcomes (53, 54).

Previous research examining these vulnerabilities simultaneously indicates that they achieve unique contributions to the prediction of psychopathology despite their intercorrelations, supporting their treatment as related but non-interchangeable transdiagnostic constructs (48). Thus, we believe that their joint examination is particularly relevant in the present population because parents of children with rare genetic disorders are repeatedly confronted both with uncertainty regarding diagnosis, prognosis, treatment, and future functioning as well as with sustained emotional distress associated with caregiving demands. Intolerance of uncertainty, worry, and distress tolerance were examined together to capture complementary vulnerability processes involving how parents appraise uncertainty, cognitively respond to anticipated threat, and tolerate the difficult emotional states generated by chronic caregiving stress.

### 1.2 The present study

Despite their established role in general and clinical populations, the contribution of transdiagnostic cognitive vulnerabilities to psychological distress in parents of children with rare genetic disorders remains insufficiently understood. Existing research has largely focused on illness-related or demographic predictors, potentially overlooking intra-individual cognitive processes that may confer differential risk for psychopathology (1, 2). Given that transdiagnostic processes are modifiable and therefore represent potential targets for intervention, it is important to examine whether they explain additional variance in parental psychological distress beyond illness-related factors.

Accordingly, the present study aimed to examine whether intolerance of uncertainty, tendency to worry, and lower distress tolerance predict symptoms of depression, anxiety, and stress beyond the severity of the child’s health status, thereby advancing a more comprehensive understanding of parental psychological distress and informing the development of targeted prevention and support interventions for parents of children with rare genetic disorders. We expected that parents of children with rare genetic disorders would report higher levels of anxiety, depression, and stress symptoms compared with healthy controls (H1). Considering that rare genetic disorders are characterized by unpredictable disease trajectories, uncertain long-term outcomes (34), as well as ongoing medical demands and disruptions in daily life, we expected that a greater severity of the child’s health problems would be positively associated with higher intolerance of uncertainty, lower distress tolerance, greater tendency to worry, and higher levels of depression, anxiety, and stress symptoms among parents (H2). Considering that individuals’ reactions to stressful events are shaped by their (mal)adaptive coping strategies and other cognitive vulnerability factors (24–26), we expected that parents’ intolerance of uncertainty, low distress tolerance, and tendency to worry would explain additional variance in depression, anxiety, and stress symptoms, above and beyond the child’s health state (H3). Finally, previous research suggests that mothers often assume a greater share of day-to-day caregiving responsibilities for children with rare or genetic disorders and are more frequently the primary caregiver (55). This greater caregiving involvement may explain in part why mothers tend to report higher levels of stress, anxiety, depression, and overall psychological distress than fathers (e.g. 18, 19). A novel contribution of this study is that it also examines gender differences in distress tolerance, intolerance of uncertainty and worry in the context of caring for children with rare genetic disorders, something which has not been explored in previous research. Thus, we expected mothers of children with rare genetic disorders to report higher levels of anxiety, depression, stress, intolerance of uncertainty, and worry, as well as lower distress tolerance, compared with fathers (H4).

## 2 Methods

### 2.1 Study sample

The total sample consisted of 278 parents. The sample consisted of two groups: the parents of children with rare genetic disorders (target group) and a comparison group comprising parents with healthy children. The sociodemographic characteristics of the sample are shown in **Table 1**. In the target group, there are 92 mother-father pairs (*n* = 184, *M_parent age_* = 37.86, *SD_parent age_* = 7.63; child age: *M_child age_* = 5.85, *SD_child age_* = 4.76). Within the target group, mothers had an average age of 35.92 years (*SD* = 6.84), while fathers had an average age of 39.8 years (*SD* = 7.92). On the other hand, the comparison group consisted of 14 mother-father pairs and 66 parents who were interviewed alone (*n* = 94, *M_parent age_* = 34.54, *SD_parent age_* = 6.76; *M_child age_* = 3.92, *SD_child age_*= 4.14). Within the comparison group, mothers had an average age of 33.7 years (*SD* = 6.38), while fathers had an average age of 35.84 years (*SD* = 7.2). No significant differences were found between parents interviewed individually and those interviewed as mother–father dyads across the examined variables.

**Table 1:** Sociodemographic characteristics of the target, control, and full samples, and health conditions of children in the target group.

Baseline measure	Target group		Control group		Full sample	
	n	%	n	%	n	%
Gender						
Female	92	50	57	60.6	149	53.6
Male	92	50	37	39.4	129	46.4
Work status						
Full-time employed	130	70.7	80	85.1	210	75.5
Part-time employed	7	3.8	6	6.4	13	4.7
Stay-at-home parent	11	6	5	5.3	16	5.8
Unemployed	26	14.1	3	3.2	29	10.4
Disabled	1	1	/	/	1	0.4
Retired	2	1.1	/	/	2	0.7
On sick leave	7	3.8	/	/	7	2.5
Monthly income						
< 426 euros	23	12.5	2	2.1	25	9
426–853 euros	87	47.3	23	24.5	110	39.6
853 – 1,279 euros	49	26.6	33	35.1	82	29.5
>1,279 euros	25	13.6	36	38.3	61	21.9
Education						
Primary school or less	9	4.9	4	4.3	13	4.7
High school	96	52.2	33	35.1	129	46.4
Associate degree	23	12.5	15	16	38	13.7
College education or higher	56	30.4	42	44.7	98	35.3
Child health problem						
Neurogenetic disorder	82	44.6	/	/	82	44.6
Congenital malformation	36	19.6	/	/	36	19.6
Inherited disorder affecting internal organs	20	10.9	/	/	20	10.9
Other rare genetic disorders	46	25	/	/	46	25

### 2.2 Procedure

The study employed a cross-sectional, questionnaire-based correlational design. Participants were consecutively recruited at the Department of Clinical Genetics, University Children’s Clinic, Belgrade. The clinical group consisted of parents of children with rare genetic disorders who agreed to participate during their child’s scheduled clinical evaluation. Based on the established diagnosis and clinical phenotype, children were classified by medical professionals into one of four groups: neurogenetic disorders, congenital malformations, inherited disorders affecting internal organs, or other rare genetic conditions. This classification is reported solely to provide a more precise description of the clinical group given the considerable heterogeneity of conditions encompassed by the term rare genetic disorders; differences between parents of children belonging to these diagnostic subgroups were not examined.

The comparison group consisted of parents of healthy children who were consecutively recruited during their children’s routine preventive visits at two primary healthcare centers. Eligible participants were parents of children without acute illness or known or suspected chronic or genetic conditions.

All participants were informed about the study procedures, and written informed consent was obtained from all participants before their inclusion in the study. The assessment battery was administered in a paper-and-pencil format during participants’ visits to the healthcare facilities, individually and in a quiet setting. After completing the questionnaires, participants were provided with additional information about the study and had the opportunity to ask questions.

The study was conducted in accordance with the principles of the Declaration of Helsinki and approved by the Ethics Committee of the University Children’s Clinic, Belgrade (Approval No. 017-16/41; July 3, 2023). Participation was voluntary and anonymous. Participants were informed of their right to withdraw from the study at any time without any negative consequences.

### 2.3 Assessment measures

A standard set of sociodemographic variables was recorded. The ages and genders of parents and children (male, female, other) were assessed, along with participants’ socioeconomic status, educational level, and employment status. Socioeconomic status was operationalized through monthly income (less than 50,000 Serbian dinars (~426 euros), 50,000 – 100,000 Serbian dinars (~426–853 euros), 100,000 – 150,000 Serbian dinars (~853 – 1,279 euros), and above 150,000 Serbian dinars (above ~ 1,279 euros)), educational level (primary school or less, high school, associate degree, college education or higher), and employment status (full-time employed, part-time employed, stay-at-home parent, unemployed, disabled, on sick leave, retired, other). Socioeconomic status, educational level, and employment status were used as covariates in the analyses.

The child’s health status severity was rated by parents using the Royal Alexandra Hospital for Children Measure of Function (RAHC MOF) scale, a generic paediatric measure of health-related functioning developed by Dossetor and colleagues (1994) and subsequently used in paediatric populations (56). The RAHC MOF comprises 10 hierarchically ordered categories, ranging from superior functioning to the need for constant medical supervision and high medical dependency. Although the instrument yields a single overall ordinal score, the criteria defining each category incorporate multiple domains of the child’s health and functioning. These include symptom severity, physical ability and mobility, emotional and behavioral functioning, social relationships, play and leisure activities, school/preschool functioning, developmental status, and the degree of medical care or supervision required. In the original validation study. parents selected the same or an adjacent MOF category as the attending clinician in 89.7% and 82.9% of cases in two outpatient samples. The authors concluded that the RAHC MOF was sufficiently reliable for comparing groups of paediatric patients (57). For additional subgroup analyses, scores of 4 or higher were considered indicative of moderate-to-severe health problems.

Regarding transdiagnostic cognitive vulnerabilities, participants’ intolerance of uncertainty, distress tolerance, and tendency to worry were assessed as antecedent variables, while depression, anxiety, and stress symptoms were assessed as outcome variables. The following instruments were used:

The Intolerance of Uncertainty Scale–Short Form (IUS; 36; Serbian version: 58) is an 11-item measure assessing the tendency to perceive uncertain situations as threatening and distressing. Answers are provided on a 5-point Likert scale, with the possible score ranging from 11 to 55. Higher scores indicate greater intolerance of uncertainty. The validated Serbian version of the scale was used (58). The scale demonstrated good reliability in the present study (average *α* = .92).

The Distress Tolerance Scale (DTS; 54) is a 15-item questionnaire assessing an individual’s perceived ability to tolerate negative psychological states. Answers are provided on a 5-point Likert scale, with the possible score ranging from 15 to 75. Higher scores indicate higher distress tolerance. The instrument was translated into Serbian and independently back-translated into English using a forward-backward translation procedure conducted by bilingual experts with C2-level English proficiency to ensure semantic equivalence with the original versions. The scale demonstrated good reliability in the present study (average *α* = .92).

The Penn State Worry Questionnaire (PSWQ; 59) is a 16-item measure assessing trait worry and the tendency to experience uncontrollable worries. Answers are provided on a 5-point Likert scale, with the possible score ranging from 16 to 80. Higher scores indicate greater levels of worry. A previous Serbian translation was used in this study (60) which was found to be highly reliable (*α* = .92). The scale demonstrated good reliability in the present study (average *α* = .9).

The Depression Anxiety Stress Scale–21 (DASS-21) (61) is a 21-item measure assessing symptoms of depression, anxiety, and stress. Answers are provided on a 4-point Likert scale, with the possible score ranging from 0 to 63. Higher scores indicate greater psychological distress. The validated Serbian version of the scale was used (62). The scale demonstrated good reliability in the present study (average *α* = .94).

### 2.4 Statistics

Differences between parents of children with rare genetic disorders and parents of healthy children were examined using ANCOVAs, controlling for socioeconomic status, educational level, and employment status. Due to the very small number of participants in the categories “disabled”, “on sick leave”, and “retired” (**Table 1**), when used as covariates they were combined with the category “unemployed” to create the category “not working”. Associations between the severity of the child’s health status severity and intolerance of uncertainty, distress tolerance, tendency to worry, depression, anxiety, and stress symptoms were examined using Pearson correlation coefficients, adjusted for multiple comparisons using the Benjamini-Hochberg false discovery rate (FDR) procedure. Hierarchical multiple regression analyses were conducted to determine whether transdiagnostic cognitive vulnerabilities accounted for additional variance in depression, anxiety, and stress symptoms beyond the severity of the child’s health status. Variance inflation factors and tolerances were calculated to test for multicollinearity. Finally, the moderating role of parent gender was examined by testing whether the associations between the severity of the child’s health status and psychological outcomes differed between mothers and fathers. In addition, gender differences were examined in the target group consisting of 92 mother–father dyads using linear mixed-effects models. Participant gender and the relevant covariates were entered as fixed effects, while a random intercept for family was included to account for the non-independence of observations originating from the same couple.

## 3 Results

### 3.1 Descriptive statistics of the study sample

Descriptive statistics of the study variables are presented in **Table 2**. All scales demonstrated high internal consistency, except for the Depression subscale in the control group, for which reliability approached an acceptable level. No particularly salient values were observed, although most measured variables showed a positive skewness. Furthermore, parents of children with rare genetic disorders consistently reported higher scores than parents of healthy children across all measured variables, indicating higher levels of depressive, anxiety, and stress symptoms, greater intolerance of uncertainty and tendency to worry, and lower distress tolerance.

**Table 2:** Descriptive statistics and internal consistency of study measures.

Instrument	Group	Min	Max	M	SD	Std.Sk	Std.Ku
Tendency to Worry	Target	24	80	49.74	13.38	1.79	-2.20
	Control	26	75	47.48	11.51	1.25	-0.92
Intolerance of Uncertainty	Target	11	55	27.01	10.12	4.29	0.41
	Control	11	43	22.80	8.21	3.38	-0.29
Distress Tolerance	Target	19	74	55.12	12.90	-3.73	-1.01
	Control	33	75	58.02	10.80	-2.29	-1.26
DASS-Depression	Target	0	18	3.74	3.87	8.14	5.56
	Control	0	12	2.00	2.28	7.20	8.41
DASS-Anxiety	Target	0	21	3.57	3.93	8.92	9.09
	Control	0	12	2.35	2.63	5.02	2.04
DASS-Stress	Target	0	21	6.80	4.45	5.61	2.85
	Control	0	14	5.74	3.24	1.35	-0.74

Min, minimal score; Max, maximum score; M, mean; SD, standard deviation; Std.SK, standardized skewness; Std.Ku, standardized kurtosis; α , Cronbach’s alpha reliability. Scores refer to raw scale scores. DASS-21 scores were analyzed in their original form.

### 3.2 Group differences

The results revealed significant differences between the target and comparison groups, with parents in the target group reporting higher levels of psychological distress. Specifically, parents in the target group reported significantly higher levels of intolerance of uncertainty (*F*(1,273) = 7.88, *p* <.05, *η2* = .03) and depressive symptoms (*F*(1,273) = 5.12, *p* <.05, *η2* = .02). The results of these analyses are presented in **Table 3**. When analyses were restricted to parents whose children experienced moderate-to-severe health problems (scores above 3 on the scale), significant differences were also observed in distress tolerance (*F*(1,151) = 3.92, *p* = .05, *η2* = .03), and stress (*F*(1,151) = 4.11, *p* <.05, *η2* = .03), in addition to intolerance of uncertainty (*F*(1,151) = 16.77, *p* <.05, *η2* = .1) and depressive symptoms (*F*(1,151) = 10.56, *p* <.05, *η2* = .07), with medium-to-large effect sizes. These results are presented in Appendix 1.

**Table 3:** Group differences in psychological distress and transdiagnostic cognitive vulnerabilities.

Measure	Target group		Comparison group		F (1, 273)	η2
	M	SD	M	SD		
Tendency to Worry	49.74	13.38	47.48	11.51	0.11	0
Intolerance of Uncertainty	27.01	10.12	22.80	8.21	7.88**	.03
Distress Tolerance	55.12	12.90	58.02	10.80	0.49	.0
Depression	3.74	3.87	2.00	2.28	5.12*	.02
Anxiety	3.57	3.93	2.35	2.63	0.55	.0
Stress	6.80	4.45	5.74	3.24	0.79	.0

Group differences between the target group (parents of children with rare genetic disorders) and the control group (parents of healthy children) were examined for each variable. M, mean; SD, standard deviation; F, F statistic; ηp², partial eta squared, *p <.05, **p <.01.

### 3.3 Associations between child health status severity, cognitive vulnerabilities, and psychological distress

Greater severity of the child’s health status was positively associated with greater tendency to worry (*r* = .18, *p* <.05), higher intolerance of uncertainty (*r* = .25, *p* <.01), and higher levels of depression (*r* = .16, *p* <.05), anxiety (*r* = .16, *p* <.05), and stress (*r* = .16, *p* <.05) (**Table 4**). Child health severity was not significantly associated with distress tolerance (*r* = −.12). As shown in **Table 4**, the three cognitive vulnerability variables were substantially intercorrelated and were also moderately to strongly associated with psychological distress.

**Table 4:** Pearson correlations among child health status and psychological variables.

	Child’s health status	Tendency to worry	Intolerance of uncertainty	Distress tolerance	Depression	Anxiety	Stress
Child’s health status	1						
Tendency to Worry	.18*	1					
Intolerance of Uncertainty	.25**	.69**	1				
Distress Tolerance	-.12	-.65**	-.74**	1			
Depression	.16*	.46**	.48**	-.55**	1		
Anxiety	.16*	.43**	.45**	-.56**	.82**	1	
Stress	.16*	.54**	.56**	-.62**	.78**	.77**	1

Pearson’s correlation coefficients are presented. Child’s health status was rated on a single-item scale, *p <.05, **p <.01. P-values for bivariate correlations were adjusted for multiple comparisons using the Benjamini–Hochberg false discovery rate (FDR) procedure.

Hierarchical multiple regression analyses showed that the severity of the child’s health condition significantly predicted depression, anxiety, and stress symptoms when entered as the sole predictor. However, its contribution became non-significant after the cognitive vulnerability variables were entered into the models (**Table 5**). All tolerance and VIF values were in the acceptable range (child’s health status (tolerance = .93, VIF = 1.08), worry (tolerance = .48, VIF = 2.1), intolerance of uncertainty (tolerance = .36, VIF = 2.75), tolerance of distress (tolerance = .42, VIF = 2.39)). With regard to depression, the proportion of explained variance increased from *R²* = .03 to *R²* = .32 (*ΔR²* = .30, *ΔF* = 26.35, *p* <.01). Lower distress tolerance was the only significant independent cognitive predictor of depressive symptoms (*β* = −.39, *t* = −4.11, *p* <.01), while tendency to worry and intolerance of uncertainty did not make significant unique contributions.

**Table 5:** Hierarchical regression analyses predicting depression, anxiety, and stress symptoms.

Outcome/Step/Predictor	R	R^2^	Adj.R^2^	Std. E	delta R^2^	F/ΔF	B	Std.E	β	t
Depression										
Step 1	0.16	0.03	0.02	3.83	0.03	4.74*				
Child’s health status							0.3	0.14	0.16	2.18*
Step 2	0.57	0.32	0.31	3.22	0.3	26.35**				
Child’s health status							0.13	0.12	0.07	1.06
Tendency to Worry							0.04	0.03	0.14	1.61
Intolerance of uncertainty							0.03	0.04	0.07	0.72
Distress Tolerance							-0.12	0.03	-0.39	-4.11**
Anxiety										
Step 1	0.16	0.03	0.02	3.89	0.03	4.83*				
Child’s health status							0.3	0.14	0.16	2.2*
Step 2	0.57	0.33	0.31	3.26	0.3	26.86**				
Child’s health status							0.15	0.12	0.08	1.29
Tendency to Worry							0.03	0.03	0.11	1.2
Intolerance of uncertainty							0.002	0.04	0.01	0.06
Distress Tolerance							-0.15	0.03	-0.48	-5.03**
Stress										
Step 1	0.16	0.02	0.02	4.41	0.02	4.5*				
Child’s health status							0.33	0.16	0.16	2.12*
Step 2	0.66	0.43	0.42	3.4	0.41	42.25**				
Child’s health status							0.09	0.13	0.04	0.71
Tendency to Worry							0.06	0.03	0.18	2.22*
Intolerance of uncertainty							0.05	0.04	0.12	1.307
Distress Tolerance							-0.14	0.03	-0.41	-4.7**

Hierarchical multiple regression analyses were conducted. Model 1 included child health status, while Model 2 additionally included worry, intolerance of uncertainty, and distress tolerance. Std. E=standard error of estimate; ΔR²=change in explained variance between models, Degrees of freedom for the first step are 1, 182, for the F change are 3, 179. *p <.05, **p <.01.

A similar pattern was observed for anxiety. Explained variance increased from *R²* = .03 to *R²* = .33 after the addition of cognitive vulnerability variables (*ΔR²* = .30, *ΔF* = 26.86, *p* <.01). Lower distress tolerance again emerged as the strongest independent predictor (*β* = −.48, *t* = −5.03, *p* <.01), while tendency to worry and intolerance of uncertainty were not independently significant.

The largest increase in explained variance was observed for stress symptoms, with *R²* increasing from.02 to.43 (*ΔR²* = .41, *ΔF* = 42.25, *p* <.01). Both lower distress tolerance (*β* = −.41, *t* = −4.70, *p* <.01) and greater tendency to worry (*β* = .18, *t* = 2.22, *p* <.05) independently predicted higher stress symptoms. Intolerance of uncertainty did not retain an independent association with stress after accounting for the other cognitive vulnerability factors. Thus, lower distress tolerance emerged as the most consistent predictor of negative emotional states.

Additional analyses examined whether the associations between cognitive vulnerability factors and psychological symptoms differed between parents of children with rare genetic disorders and parents of healthy children. A significant moderating effect of group was observed, indicating that the associations of tendency to worry and lower distress tolerance with depression, anxiety, and stress symptoms were stronger among parents of children with rare genetic disorders than among parents of healthy children (**Table 6**).

**Table 6:** Differences in the predictive value of transdiagnostic cognitive vulnerabilities for psychological distress across study groups.

Target group	Outcome/Predictor	R	Adj.R^2^	Std. E	F (3, 180)	B	Std.E	β	t
	Depression	0.57	0.31	3.22	28.2**				
	Tendency to Worry					0.04	0.03	0.15	1.66
	Intolerance of uncertainty					0.04	0.04	0.09	0.94
	Distress Tolerance					-0.11	0.03	-0.38	-4.02**
	Anxiety	0.57	0.31	3.27	28.5**				
	Tendency to Worry					0.03	0.03	0.11	1.26
	Intolerance of uncertainty					0.01	0.04	0.03	0.32
	Distress Tolerance					-0.14	0.03	-0.46	-4.91**
	Stress	0.65	0.42	3.4	44.72**				
	Tendency to Worry					0.06	0.03	0.18	2.26*
	Intolerance of uncertainty					0.06	0.04	0.14	1.48
	Distress Tolerance					-0.14	0.03	-0.4	-4.66**
Comparison group	Outcome/Predictor	R	Adj.R2	Std. E	F (3, 90)	B	Std.E	β	t
	Depression	0.46	0.19	2.06	8.09**				
	Tendency to Worry					-0.03	0.03	-0.15	-1.19
	Intolerance of uncertainty					0.06	0.05	0.21	1.33
	Distress Tolerance					-0.08	0.03	-0.35	-2.60*
	Anxiety	0.4	0.13	2.45	5.71**				
	Tendency to Worry					0.04	0.03	0.19	1.5
	Intolerance of uncertainty					0.004	0.05	0.01	0.07
	Distress Tolerance					-0.06	0.03	-0.26	-1.86
	Stress	0.49	0.21	2.88	9.37**				
	Tendency to Worry					0.08	0.04	0.29	2.34*
	Intolerance of uncertainty					0.01	0.06	0.03	0.22
	Distress Tolerance					-0.08	0.04	-0.25	-1.86

Separate multiple regression analyses were conducted for the target and comparison groups to examine differences in predictive strength of cognitive vulnerability factors. Predictors included worry, intolerance of uncertainty, and distress tolerance. Std. E=standard error of estimate. *p <.05, **p <.01.

### 3.4 Moderating role of gender

An expected moderating effect of gender was observed, whereby correlations between the child’s health state and psychological variables were stronger among mothers of children with developmental issues in comparison to fathers. This suggests that as the child’s health status worsens, the mental state of both parents worsens as well, although among mothers to a greater degree in comparison to fathers. The relevant correlations can be seen in Appendix 2. Furthermore, the results of mixed linear model analysis suggest that mothers fared substantially worse compared to fathers in the target group, scoring higher on worry (EMM = 51.56 vs. 45.87), *F*(1, 94.66) = 13.88, *p* <.001, *η^2^* = .128, intolerance of uncertainty (27.61 vs. 24.16), *F*(1, 92.7) = 11.73, *p* = .001, *η2* = .112, depression (3.64 vs. 2.55), *F*(1, 94.89) = 5.53, *p* = .021, *η2* = .055, anxiety (3.94 vs. 2.61), *F*(1, 91.28) = 8.73, *p* = .004, *η2* = .086, and stress (7.27 vs. 5.52), *F*(1, 92.3) = 10.45, *p* = .002, *η2* = .102, while scoring lower on distress tolerance (53.24 vs. 56.83), *F*(1, 93.59) = 6.45, *p* = .013, *η^2^* = .064. The results of the relevant analyses can be seen in **Table 7**.

**Table 7:** Gender differences in psychological outcomes based on linear mixed-effects models in the target group.

Measure	Mothers EMM (SE)	Fathers EMM (SE)	Adjusted difference B [95% CI]	F(df1,df2)	η2
Tendency to Worry	51.56 (2.00)	45.87 (2.30)	5.69 [2.66, 8.72]	13.88** (1, 94.66)	0.13
Intolerance of Uncertainty	27.61 (1.47)	24.16 (1.66)	3.44 [1.45, 5.44]	11.73** (1, 92.7)	0.11
Distress Tolerance	53.24 (1.93)	56.83 (2.21)	-3.6 [-6.41, -0.78]	6.45* (1, 93.59)	0.06
Depression	3.64 (0.58)	2.55 (0.68)	1.09 [0.17, 2]	5.53* (1, 94.89)	0.06
Anxiety	3.94 (0.58)	2.61 (0.67)	1.33 [0.44, 2.22]	8.73** (1, 93.28)	0.09
Stress	7.27 (0.68)	5.52 (0.79)	1.76 [0.68, 2.83]	10.45** (1, 92.3)	0.1

EMM, estimated marginal mean; B represents the adjusted mother–father difference. Models controlled for education, income, and employment status and included a random intercept for couple. Partial η^2^ values were derived from the fixed-effect F statistics. *p <.05, **p <.01.

**Table 8:** Potential intervention targets for parents of children with rare genetic disorders. A detailed overview of possible interventions is presented in **Table 8**.

Intervention focus	Targeted need	Measures	Evidence base
Reducing caregiving burden	High practical and illness-related demands	Care coordination; medical, social, and financial support; rehabilitation; respite care; genetic counselling	Present cross-sectional findings; observational studies and systematic reviews of rare-disease caregiving (11, 63)
Distress-tolerance intervention	Difficulty tolerating persistent negative emotions	CBT- and DBT-informed emotion-regulation and distress-tolerance skills; exposure to difficult internal experiences; individual, group, or online delivery	Present cross-sectional findings; evidence from a brief transdiagnostic distress-tolerance intervention, a systematic review and meta-analysis of DBT skills training, and preliminary evidence that CBT may improve distress tolerance (64–66); limited direct evidence in rare-disease caregivers
Worry-focused intervention	Repetitive worry about prognosis, treatment, and the child’s future	Psychoeducation; cognitive restructuring; behavioral experiments; exposure to uncertainty; reduction of reassurance seeking	Present cross-sectional findings; meta-analytic and theoretical evidence supporting worry and intolerance of uncertainty as related but distinguishable treatment targets (50, 51)
Integrated stepped care	Combined practical burden and psychological vulnerability	Screening followed by psychoeducation, online support, targeted CBT, or specialist referral according to need	Synthesis of present findings and external evidence; rare-disease RCT and broader systematic review and meta-analysis of online interventions (67, 68)

## 4 Discussion:

### 4.1 Principal findings and theoretical implications

The aim of this study was to examine whether parental transdiagnostic cognitive vulnerabilities- intolerance of uncertainty, distress tolerance, and tendency to worry contribute to the explanation of depression, anxiety, and stress symptoms beyond the severity of the child’s health condition in parents of children with rare genetic disorders. More broadly, we tested a dual explanatory framework of parental distress, distinguishing between illness-related factors (i.e., the severity of the child’s condition) and transdiagnostic psychological vulnerabilities that may shape how parents respond to chronic stress. To the best of our knowledge, this is the first study to integrate these two perspectives within a heterogeneous sample of parents of children with rare genetic disorders, addressing a literature that has largely relied on disorder-specific findings with limited generalizability.

Results showed that parents of children with rare genetic disorders reported higher levels of depression, anxiety, and stress symptoms than parents of healthy children. They also reported greater intolerance of uncertainty and lower distress tolerance, suggesting that the experience of caring for a child with a rare genetic disorder is associated not only with increased emotional distress but also with specific cognitive and emotional vulnerabilities. These differences were particularly evident among parents whose children had moderate-to-severe health conditions, consistent with previous research identifying disease severity as an important contextual stressor (1, 2, 10). Recent reviews similarly indicate that parenting a child with a rare disorder is associated with multifaceted psychological and social burdens, with uncertainty and the unknown emerging as central features of parents’ experience (69).

Greater severity of the child’s health condition was associated with poorer parental mental health, and this pattern was particularly evident among mothers. This finding is consistent with evidence showing that mothers often continue to carry a disproportionate share of caregiving responsibilities (17, 21). In the present study, mothers reported a greater tendency to worry, intolerance of uncertainty, depression, anxiety, and stress, as well as lower distress tolerance than fathers, suggesting that their greater vulnerability was not limited to a single aspect of psychological functioning. These obtained differences may partly reflect the greater day-to-day caregiving burden experienced by mothers, together with the broader physical, occupational, and financial consequences of caring for a child with a rare genetic condition (22, 23). These findings therefore suggest that both illness-related factors and gendered caregiving roles contribute to parental distress but do not fully explain the considerable variability observed among parents.

The key contribution of the present study is the finding that transdiagnostic cognitive vulnerabilities explained additional variance in parental psychological distress beyond illness severity. Although the severity of the child’s health condition significantly predicted depression, anxiety, and stress symptoms in the initial models, its contribution was substantially reduced after psychological vulnerabilities were included. The inclusion of these factors increased the explained variance from approximately 3% to more than 30%, highlighting their relevance for understanding parental distress. Among the examined vulnerabilities, lower distress tolerance emerged as the strongest and most consistent independent correlate of depressive, anxiety, and stress symptoms. Greater tendency to worry also showed an additional contribution, particularly for stress symptoms, after accounting for the severity of the child’s health condition.

These findings suggest that parental distress is associated not only with the objective burden imposed by the child’s condition but also with individual differences in how parents experience and regulate difficult emotional states. Similar levels of caregiving demands may therefore be associated with different psychological outcomes depending on parents’ ability to tolerate distress and the extent to which tendency to worry becomes a dominant cognitive response. Although causal conclusions cannot be drawn from the present cross-sectional design, the observed pattern is consistent with the view that transdiagnostic cognitive vulnerabilities represent important psychological factors associated with parental distress.

One possible explanation for the prominent role of distress tolerance is that uncertainty represents only one aspect of the caregiving experience, whereas distress tolerance reflects a broader capacity to endure and regulate a wide range of aversive emotional states, including fear, sadness, frustration, exhaustion, and helplessness. This interpretation is consistent with transdiagnostic models conceptualizing distress tolerance as an important vulnerability factor influencing responses to emotional distress (53). The present findings extend this literature by suggesting that distress tolerance may be particularly relevant for psychological adjustment among parents of children with rare genetic disorders, who often face persistent emotional demands extending beyond uncertainty alone.

Although intolerance of uncertainty did not remain an independent predictor after accounting for shared variance with other cognitive vulnerabilities, it was elevated among parents of children with rare genetic disorders. This finding is theoretically meaningful given the prolonged uncertainty surrounding diagnosis, prognosis, treatment possibilities, and future development in this population. Previous research has consistently linked intolerance of uncertainty with anxiety in situations characterized by unpredictability (30, 70). Its role in parents of children with rare genetic disorders therefore remains an important area for further investigation. Contemporary transdiagnostic accounts further support this interpretation by conceptualizing intolerance of uncertainty as a relatively distal vulnerability and worry as a more proximal cognitive process rather than as redundant constructs (50, 51).

Taken together, these findings suggest that it is not only the amount of stress parents experience but also how they respond to that stress that is associated with psychological outcomes. While illness severity defines the context of caregiving demands, transdiagnostic cognitive vulnerabilities may influence how these demands are interpreted and managed. This perspective extends previous research that has predominantly emphasized disease-related characteristics and supports a more comprehensive understanding of parental distress that integrates both contextual and individual psychological factors.

This interpretation is particularly relevant given the broader clinical context of rare genetic disorders. Only a small proportion of these conditions are causally treatable, with most disorders managed through symptomatic approaches (71). Consequently, parents often face prolonged uncertainty, unclear prognoses, and continuous decisions regarding treatment and care. These challenges are further intensified by demanding caregiving responsibilities and ongoing developmental difficulties (72, 73). At the same time, parents play a central role in implementing developmental interventions (74, 75), and parental mental health has consistently been associated with child outcomes (76–79). Therefore, addressing parental psychological functioning may have implications not only for parents themselves but also for family adaptation and child development. Although genome and exome sequencing, long-read sequencing, and complementary multi-omics approaches have substantially expanded the diagnostic possibilities for rare genetic diseases, many families still encounter unresolved findings, variants of uncertain significance, and limited prognostic or therapeutic certainty (80). Thus, contemporary genomic advances may provide relief with regard to the sources and timing of uncertainty without wholly eliminating the psychological burden associated with it.

The present findings are also consistent with broader transdiagnostic models of emotional disorders. Lower distress tolerance has been associated with a wide range of psychological difficulties (46–48). Similarly, worry reflects repetitive and difficult-to-control negative thinking that has been linked to emotional distress (36, 37), while repetitive negative thinking represents a shared vulnerability underlying both anxiety and depressive symptoms (40). More generally, transdiagnostic models emphasize maladaptive responses to internal experiences as common processes underlying multiple forms of psychopathology (25, 81). In the context of persistent caregiving demands, these processes may represent important pathways linking chronic stress exposure with psychological distress.

From a theoretical perspective, the present findings extend the application of transdiagnostic models of emotional disorders to the context of chronic parental stress. Specifically, they suggest that variability in parental distress is shaped not only by objective caregiving demands but also by individual differences in core cognitive vulnerabilities. By integrating illness-related and psychological factors within a single framework, this study provides a more comprehensive account of parental distress and moves beyond models focused primarily on disease severity. Overall, the findings indicate that although caregiving demands define the context in which stress occurs, transdiagnostic cognitive vulnerabilities- particularly lower distress tolerance play an important role in how parents experience and respond to chronic stress.

### 4.2 Clinical implications

These findings also have important clinical implications. Early identification of parents with lower distress tolerance and elevated worry may help identify those who could benefit from psychological support in pediatric and genetic healthcare settings. Routine psychological screening could facilitate timely referral to appropriate interventions.

Interventions targeting these transdiagnostic processes may represent promising approaches for supporting parents of children with rare genetic disorders. Cognitive-behavioral therapy (CBT), which has strong empirical support for anxiety and depression (82–85), includes techniques relevant to difficulties in tolerating distress and maladaptive worry, such as cognitive restructuring, behavioral experiments, and exposure-based approaches. Dialectical behavior therapy (DBT), a cognitive-behavioral treatment emphasizing skills acquisition, may be particularly relevant to low distress tolerance through its distress-tolerance and emotion-regulation modules. Evidence from a recent systematic review and meta-analysis supports the use of DBT skills training across common mental health problems (64). Because these approaches address underlying psychological processes rather than disorder-specific symptoms alone, they may provide broader benefits for parental psychological functioning. Improving parental well-being may also support caregiving capacity and family adaptation. Given the challenges of providing specialized psychological support to families affected by rare genetic disorders, scalable approaches such as group-based and internet-delivered CBT and DBT-informed skills training may further improve access to evidence-based interventions (S. 86–88). This emphasis is consistent with recent calls for psychological support to be integrated into rare-disease care, particularly around diagnosis and other periods of heightened uncertainty (63).

### 4.3 Limitations

Although this study contributes to the literature in several ways, a number of limitations should be acknowledged. First, the cross-sectional design does not allow for conclusions about causality, and longitudinal research would be needed to clarify the direction of effects between cognitive vulnerabilities and parental distress. Second, all variables were assessed using self-report measures, which may introduce bias; therefore, future studies could benefit from including clinician-rated or multi-informant assessments. Third, the assessment of the child’s health status relied on a single parent-provided measure, and consequently, the score may reflect both the child’s actual health-related functioning and the respondent’s perception of the child’s condition. Thus, future research could incorporate more detailed indicators of illness severity, functional impairment, and caregiving burden. Fourth, gender comparisons were restricted to the target group consisting of families in which data were available from both mothers and fathers, considering that the control group was structurally imbalanced with substantially greater representation of mothers. Finally, the sample was heterogeneous with regard to the child’s condition. While this limits disorder-specific conclusions, it can also be seen as a strength, as it allows for the examination of transdiagnostic processes across a broader range of rare genetic disorders.

## 5 Conclusion

Parents of children with rare genetic disorders reported higher levels of depression, anxiety, stress symptoms, and transdiagnostic cognitive vulnerabilities compared with parents of healthy children. Parental psychological distress was independently associated with lower distress tolerance and greater tendency to worry beyond the severity of the child’s health condition. These findings highlight the importance of targeting transdiagnostic cognitive processes in psychological interventions for parents, with potential benefits for parental well-being and caregiving functioning.

## Data Availability

The raw data supporting the conclusions of this article will be made available by the authors, without undue reservation.

## APPENDIX

### Appendix 1 Group differences between parents of children with moderate-to-severe health status and parents of healthy children

**Table:**

Measure	Target group		Comparison group		F (1, 151)	η2
	M	SD	M	SD		
Tendency to Worry	52.39	14.26	47.48	11.51	2.5	.02
Intolerance of Uncertainty	29.77	9.71	22.8	8.21	16.77**	.1
Distress Tolerance	53.13	11.99	58.02	10.80	3.92*	.03
Depression	4.65	4.27	2.00	2.28	10.56**	.07
Anxiety	4.40	4.49	2.35	2.63	2.92	.02
Stress	7.85	4.98	5.74	3.24	4.11*	.03

Group differences between comparison group and parents of children with moderate-to-severe health status was examined using one-way ANCOVAs. M=mean; SD=standard deviation; η² = eta squared. *p <.05, **p <.01.

## Appendix 2 Correlations between child health status, transdiagnostic cognitive vulnerabilities, and psychological distress between mothers and father separately

**Table:** .

	Child’s health	Tendency to worry	Intolerance of uncertainty	Distress tolerance	Depression	Anxiety	Stress
Child’s health	1	.13	25*	-.14	.14	.14	.09
Tendency to Worry	.23*	1	.69**	-.72**	.53**	.49**	.54**
Intolerance of Uncertainty	.25*	.67**	1	-.81**	.50**	.47**	.53**
Distress Tolerance	-.1	-.57**	-.66**	1	-.51**	-.57**	-.55**
Depression	.18	.39**	.44**	-.56**	1	.86**	.78**
Anxiety	.18	.35**	.41**	-.53**	.79**	1	.78**
Stress	.21*	.50**	.56**	-.66**	.77**	.75**	1

Pearson’s correlation coefficients are presented. Child’s health status was rated on a single-item scale, *p <.05, **p <.01. Correlations for mothers are presented below the diagonal; correlations for fathers are presented above the diagonal. P-values for bivariate correlations were adjusted for multiple comparisons using the Benjamini–Hochberg false discovery rate (FDR) procedure.
